# The Mental Health Implications of Corporate Social Responsibility: The Significance of the Sense-Making Process and Prosocial Motivation

**DOI:** 10.3390/bs13100870

**Published:** 2023-10-23

**Authors:** Byung-Jik Kim, Min-Jik Kim, Dong-gwi Lee

**Affiliations:** 1 Department of Psychology, Yonsei University, Seoul 03722, Republic of Korea; kimbj8212@ulsan.ac.kr; 2 College of Business, University of Ulsan, Ulsan 44610, Republic of Korea; 3 School of Industrial Management, Korea University of Technology and Education, Cheonan 1600, Republic of Korea

**Keywords:** corporate social responsibility, depression, meaningfulness of work, prosocial motivation, moderated mediation model

## Abstract

As corporate social responsibility (CSR) has progressively ascended to prominence among academics and industry professionals, numerous studies have embarked on examining its impact on employees’ perceptions, attitudes, and behaviors. Notwithstanding, the current body of research has predominantly overlooked the influence of CSR on employees’ mental health, encompassing depression, anxiety, and burnout. In order to acknowledge the critical role of employee mental health within an organization, our exploration is focused on discerning the effect of CSR on depressive states. Furthermore, our paper undertakes a thorough analysis of the link between CSR and depression, probing its underlying processes and potential contingent factors. We posit that CSR can alleviate the incidence of employee depression by amplifying the sense of meaningfulness that work provides. Moreover, the element of prosocial motivation among employees may act as a positive moderating variable that intensifies the beneficial effect of CSR on the sense of meaningfulness derived from work. By relying on data obtained through a tripartite online survey involving 214 South Korean workers, this paper scrutinized the proposed hypotheses via the application of moderated mediation analysis with structural equation modeling. We contend that the insights yielded by this study bear significant theoretical and practical implications.

## 1. Introduction

Within the past several decades, the notion of corporate social responsibility (CSR) has surged in relevance among both academics and industry professionals. Despite the multitude of definitions and interpretations assigned to the core principle of CSR, there exists a broad scholarly consensus that it encapsulates a range of strategies and policies oriented toward fostering economic, social, and environmental advancement by addressing the requirements of a diverse spectrum of stakeholders, encompassing employees, customers, local communities, and the environment [1]. Accumulated evidence attests to the efficacy of CSR as a strategy to enhance a corporation’s competitive standing [2,3] by fortifying its corporate image or prestige, amplifying consumer appraisal of the firm and its product range, elevating its attractiveness to potential investors, and improving financial outcomes [2,4,5,6].

Specifically examining the employee’s responses to CSR initiatives, research has unveiled that CSR initiatives could potentially augment the quality of employees’ perceptions, attitudes, and behaviors. These include increased identification with the organization, employee engagement, a sense of meaningful work, creativity, innovative behavior, and organizational citizenship behavior [2,7,8,9,10].

Even though a wealth of literature on CSR has explored the effects of corporate social responsibility on various organizational outcomes, we posit that there still exist voids in the research landscape that merit attention. Primary among these is the role of employees’ mental health in the workplace, such as manifestations of burnout, depression, and anxiety, which has been relatively overlooked in previous CSR research. With regard to our current understanding, there has been a dearth of work investigating the impact of corporate social responsibility on the mental health of employees at work [2,3,8,11]. Given the substantial impact of an employee’s mental health on their workplace perceptions, attitudes, and behaviors, either directly or indirectly [12,13,14,15,16,17], it becomes essential to examine this relationship [2,8,11]. 

Second, the association between CSR and the mental health of employees is an area that remains inadequately explored, with limited attention being paid to the intermediary processes (or mediators) and contextual factors (or moderators) of this relationship [2,8,11]. The comprehension of these underlying mechanisms and contingent factors would provide a more nuanced understanding, enable prediction, and even allow for control over the relationships, thereby rendering an examination of these mediators and moderators valuable.

Third, the significance of an employee’s interpretative process in reacting to an organization’s CSR endeavors is an aspect that has been relatively neglected within the existing literature [2,8,18]. Given that individual employees maintain unique interpretative processes or perceptions, which are likely influenced by personal characteristics or circumstances, uniform responses to CSR initiatives across all employees cannot be assumed [18,19,20]. As these interpretative processes or perceptions can shape employees’ views and attitudes towards CSR initiatives [18,20], it becomes crucial to examine the various factors related to sense-making, such as meaningful work and prosocial motivation, within the context of CSR literature.

Fourth, extant studies have paid inadequate attention to mediating or moderating aspects of employees’ positive psychology, such as meaningful work, forgiveness, gratitude, and prosocial motivation when trying to elucidate the underlying mechanisms and environmental factors linking CSR and multiple organizational outcomes [2,8,21]. The field of positive psychology strives to explain various organizational phenomena via the lens of positive procedures and outcomes, as opposed to their negative counterparts [21]. Put differently, past research has predominantly concentrated on the ‘negative’ facets of organizational life. However, considering that ‘real-world’ organizations embody both positive and negative elements, it is important to explore these underlying mechanisms and contingent variables from a positive psychology perspective. 

In order to address the research gaps, our investigation aims to investigate the influence of corporate social responsibility on employee depression levels, utilizing an approach rooted in positive psychology. Specifically, we concentrate on the intermediary role of meaningful work [15,16,17,18,19,20,22,23,24], coupled with the moderating influence of prosocial motivation [25,26,27]. These factors are recognized as representing the sense-making processes of an organization’s workforce [18,19,28].

We anticipate that the current paper will provide significant contributions to both the CSR and depression literature for several reasons. First, we seek to uncover the connection between CSR and employees’ mental health (specifically, depression), investigating the mediating influence of meaningful work and the contextual role of prosocial motivation. Secondly, to illuminate the mediating and moderating elements within the CSR-depression association, this manuscript underscores the crucial function of employees’ sense-making processes (namely, meaningful work and prosocial motivation) in reacting to their organization’s CSR initiatives. Thirdly, we scrutinize the mediation process and its contextual variable within the CSR-depression nexus from a positive psychological standpoint. Lastly, methodologically, we aim to address the limitations of the cross-sectional research by deploying three-wave time-lagged data collected from 214 South Korean workers.

## 2. Theory and Hypotheses

### 2.1. CSR and Depression

The current paper proposes that CSR initiatives may lead to a reduction in employees’ levels of depression or depressive symptoms. Despite limited investigations directly probing this relationship, our hypothesis is formulated based on the existing literature, encompassing both CSR and depression. Meta-analytical research reveals that approximately one-fifth of the world’s population has experienced symptoms of depression or full-blown depressive disorders [16,29,30]. Depression acts as a significant precursor to diminished psychological adjustment and is a widespread, serious health issue [16]. Additionally, it is associated with deteriorating social relationships [31] and general health issues [32,33].

In terms of relevancy to our context (i.e., workplace), depression is known to be one of the most common mental illnesses at work [12,13]. There is a negative correlation between employees’ work performance [12,34], occupational functioning, absenteeism, and productivity [14,15,16,17]. 

Furthermore, insights from the CSR literature allow us to hypothesize that CSR may alleviate employees’ depression or depressive symptoms. Existing CSR research has indicated that CSR initiatives boost employees’ ‘positive’ perceptions, attitudes, and behaviors, including job psychological well-being, satisfaction at work, identification with their organization, work engagement, and prosocial behavior [2,7,8,9,10,35]. Such positive perceptions and attitudes elicited by CSR activities have been found to reduce depression levels [15,16,23,24]. Given these premises, we propose the subsequent hypothesis.

**Hypothesis** **1.** *Employee perceptions of CSR are negatively related to employee depression or depressive symptoms*.

### 2.2. CSR and Meaningfulness of Work

We posit that CSR initiatives amplify the sense of meaningful work experienced by employees [18,19,20,28]. The concept of meaningful work is multifaceted, encompassing an individual’s overarching beliefs, values, and attitudes toward their work to the psychological significance and experiential aspects of their occupation [19,20,36]. Within an organizational context, it substantially impacts employees’ interpretations of diverse workplace events, their experiences within the organization, and their approach to task execution [20,28]. Consequently, the meaningfulness of work plays a pivotal function in shaping members’ attitudes and behaviors at work [19,20].

Of the multitude of methods used to augment the meaningfulness of work, making societal contributions stands out as a particularly effective strategy [37,38,39]. An employee who perceives their work as a vehicle for enhancing others’ happiness and effecting societal improvements may experience a heightened sense of meaningful work within their organization [36,37,38,39]. From the perspective of employees, their active or indirect participation in CSR initiatives through the fulfillment of their roles offers a potent and efficient means to contribute to society. This, in turn, is likely to foster a sense of meaningfulness in their work [18]. In light of the aforementioned discussion, this paper suggests the ensuing hypothesis.

**Hypothesis** **2.** *Employees’ perception of CSR will be positively related to employees’ meaningfulness at work.*

### 2.3. Meaningfulness of Work and Depression

The current research suggests that the meaningfulness members find in their work diminishes their levels of depression. While minimal studies have delved into the impact of meaningful work on depression, we can anticipate an association between these constructs that is grounded in previous investigations of meaningful work and depression. The meaningfulness of work has been known to possess a significant correlation with key ‘positive’ employee perceptions and attitudes, such as satisfaction at work, employee engagement, organizational commitment, and psychological well-being, as well as ‘negative’ ones, including job stress and burnout [19,20,22].

An employee who finds a high degree of meaningfulness in his or her work may experience positive psychological states over negative ones. These positive employee perceptions and attitudes, which are a consequence of meaningful work, are recognized to lessen the severity of depressive symptoms and depression [15,16,23,24]. On the basis of the considerations, the paper suggests the subsequent hypothesis.

**Hypothesis** **3.** *An employee’s meaningfulness at work will be negatively related to their depression.*

### 2.4. Mediating Effect of Meaningful Work in the CSR-Depression Link

Drawing on the theoretical scaffolding provided by the preceding discussions, our argument proposes that the construct of the ‘meaningfulness of work’ experienced by employees operates as a mediating factor within the context of the impact of CSR on depression. This claim is firmly anchored in established theoretical foundations that underscore the capacity of CSR to augment the level of meaningfulness that employees perceive in their work [18,19,20].

Our argument suggests that the elevated sense of meaningfulness consequent to CSR initiatives is likely to manifest as a reduction in the prevalence of depressive symptoms among employees. This inference is substantiated by a significant body of research that underscores the beneficial effects of meaningful work in mitigating the severity of depressive symptoms [15,16,23].

Conceiving these hypotheses as part of an integrative theoretical framework enhances our understanding of these interactions within the organizational milieu. Therefore, we postulate the following mediation hypothesis, which is firmly rooted in our comprehensive research model. This enriched theoretical framework does not merely attempt to confirm separate associations but aims to elucidate the complex interplay among these constructs. By pursuing this comprehensive and synergistic theoretical approach, we amplify the robustness of our argument, thereby laying a solid foundation for consequential empirical examinations.

**Hypothesis** **4.** *Employees’ meaningfulness at work will mediate the link between employees’ perceptions of CSR and depression.*

### 2.5. Moderating Effect of Employee Prosocial Motivation in the Link between CSR and Meaningfulness of Work

Our argument posits that the degree of prosocial motivation amongst employees could potentially moderate the association between CSR and the meaningfulness of work. While acknowledging the presence of theoretical and empirical studies that endorse the link between CSR and the meaningfulness of work [18,19,20,22], we argue that a universal assumption of the enhancing effect of CSR on meaningful work may oversimplify the actual dynamics within organizational settings. There may exist numerous contextual variables that directly or indirectly shape this relationship.

By focusing on the myriad of potential moderating factors, our attention is directed toward employees’ prosocial motivation, which is characterized as the innate demand to defend and enhance the well-being of other people in need [40]. This form of motivation captures the employee’s motivational qualities, which are intimately connected to the sense-making process by significantly influencing their perceptions, attitudes, and behaviors [26,41,42].

Given the propensity of an organizational member to make sense of his or her experiences at work and engage proactively in his or her organization, it is reasonable to hypothesize that employees do not passively comply with the company’s CSR activities. Instead, they rely on their sense-making interpretations to decide how to respond and collaborate with the firm’s ethical endeavors (i.e., CSR). We, therefore, hypothesize that prosocial motivation can considerably affect how employees comprehend the processes and interpret the organization’s CSR initiatives, ultimately shaping their reactions (e.g., perceptions, attitudes, and behaviors) toward CSR activities.

In particular, we posit that prosocial motivation can play a beneficial role as a moderating variable, which amplifies the positive effect of CSR on meaningful work. In accordance with the theory of person-organization fit [43,44,45], a synergistic dynamic may emerge when an individual’s value framework aligns with their employing organization’s values. For instance, employees displaying a strong inclination towards prosocial motivation may tend to endeavor to make a societal impact through their occupational engagements. As a result, these individuals will be more inclined to perceive an alignment between their personal values and the ethical actions taken by the organization (i.e., CSR) [25]. This alignment would lead them to react positively to the CSR activities of their organization. Then, such employees may perceive their organization’s CSR initiatives as fulfilling their ethical obligations to contribute to societal well-being [25,26,27]. Consequently, they are more likely to interpret CSR efforts favorably, experiencing an enhanced sense of meaningful work.

On the contrary, members with a low degree of prosocial motivation are less likely to resonate with the moral values and actions embodied by their organization. In this situation, such employees might be reluctant to engage with the CSR initiatives, downgrading the ethical endeavors of their organization. These individuals might be apathetic or even react negatively to the CSR initiatives as they might perceive these activities as an inconsequential ‘expense’ [25,26]. In such scenarios, the positive impact of corporate social responsibility on the sense of meaningfulness would be diminished.

**Hypothesis** **5.** *Employees’ prosocial motivation will positively moderate the link between employees’ perceptions of CSR and meaningfulness at work*.

**Figure 1:**
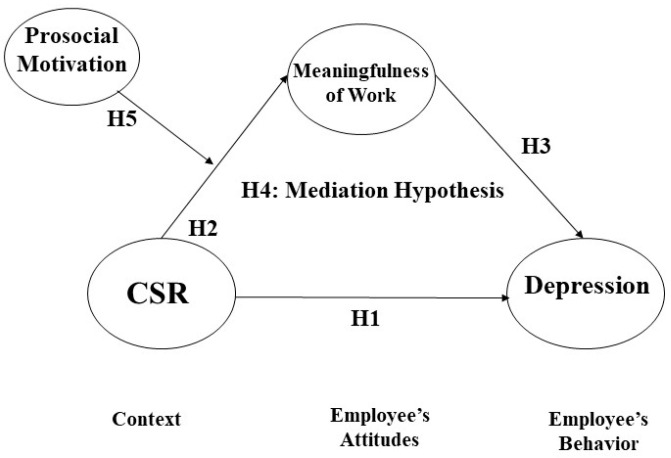
Theoretical model of this study.

Figure 1 illustrates our theoretical model and all six hypotheses formulated in this section.

## 3. Research Methodology

### 3.1. Participants and Procedure 

The research population encompassed working professionals aged 20 and above hailing from various organizations within South Korea, spanning across three distinct time frames. Their recruitment was achieved via an online survey agency boasting the most extensive research panelist database (approximately 4,180,000) within their online survey system. The respondents disclosed their employment status while enrolling for the online membership through a user authentication procedure (either through their mobile number or email address). Such online survey platforms are widely recognized as reliable avenues for acquiring diverse samples [46].

The data acquisition process was undertaken by the employees of South Korean companies at three separate intervals. This methodology was deployed to overcome the inherent challenges associated with cross-sectional research design. The research agency allocated a unique identifier to each respondent, managed through the company’s online survey infrastructure. This facilitated the accurate matching of questionnaires across the three stages. The system’s operational features permitted us to observe the respondents’ activities, thereby ensuring consistency in the participant pool from the initial to the final stage. The interlude between the initial and second survey ranged from 4 to 5 weeks, while the gap between the second and final survey varied between 12 and 13 weeks. The differential temporal gaps between T1-T2 and T2-T3 can be attributed to the longer manifestation period of CSR impact on an employee’s depression relative to its influence on the employee’s ‘attitudes’ [2,8,10,15,16]. The survey system was available for a period of two to three days during each phase to allow respondents ample time for completion. Respondents had the liberty to access the system at any point while it was operational. In order to maintain data integrity, geo-IP violators were flagged, and timestamps were utilized to identify speedy responses, thereby prohibiting respondents from repeatedly logging onto the survey portal and submitting multiple responses. 

The survey respondents were directly approached by the research agency to obtain their voluntary participation consent. This approach also ensured the strict confidentiality of their responses, which were earmarked exclusively for research objectives. Additionally, the organization gathered and documented informed consent and compliance with ethical guidelines from those agreeing to participate. The participants were remunerated with a monetary reward (USD 8–9) for their contribution. The study received formal approval from the Institutional Review Board of a prominent South Korean university.

In order to mitigate sampling bias, the research agency employed stratified random sampling for participant selection. In this sampling method, a random sample is sourced from each required stratum. This approach curtails any potential bias stemming from diverse employee attributes (such as gender, age, position, education, and industry type) that could potentially skew the research findings. Owing to the advanced operational functions of the online system, we were able to monitor the respondents, ensuring consistent participation from the first to the third time points.

The initial survey time point witnessed the participation of 408 employees, followed by 289 and 221 in the subsequent second and third time points, respectively. After data collection, the responses with missing data were removed. Ultimately, data from 214 employees providing comprehensive responses across the three-phase survey were utilized (response rate: 52.45%). In order to ascertain the sample size, we referred to multiple prior studies. Initially, we employed G*Power version 3.1.9.7 to compute the minimum sample size required to validate our study. Power analysis revealed that a sample size of 214 ensured sufficient power (≥0.70) to detect a medium effect at an alpha level of *p* = 0.05 [47]. The characteristics of the respondents are represented in Table 1.

### 3.2. Measures

The administered questionnaire evaluated discrete variables encapsulated within our research model at each designated time interval. At the initial time frame, the respondents were solicited for their perceptions regarding CSR and prosocial motivation. During the second time frame, data were collated from participants to assess the extent of their perceived meaningfulness in their work. During the third and final time frame, information was gathered regarding the participants’ experience of depression. These variables were gauged via multi-item scales by utilizing a five-point Likert scale, where 1 represents strong disagreement, and 5 indicates strong agreement. Additionally, the internal consistency of each variable was ascertained through the computation of Cronbach’s alpha values. 

#### 3.2.1. CSR (Time Point 1, Collected from Employees)

The extent of corporate social responsibility within each organization was quantified utilizing the 12-item scale devised by Turker [48] for assessing CSR. This metric was formulated by adopting a stakeholder perspective that emphasizes CSR endeavors dedicated to a diverse set of stakeholders. In this study, we opted for four distinct facets of CSR initiatives: environmental stewardship, community involvement, employee welfare, and customer relations. Each of these four dimensions comprised three items. In order to illustrate this, for the environmental stewardship dimension, a representative item was “our company participates in activities which aim to protect and improve the quality of the natural environment”. Regarding the community dimension, the sample item was “our company contributes to campaigns and projects that promote the well-being of the society”. Regarding the employee dimension, the sample item was “The management of our company is primarily concerned with the employees’ needs and wants”. Regarding the customer dimension, the sample item was “our company respects consumer rights beyond the legal requirements”. The Cronbach’s alpha value is 0.90.

#### 3.2.2. Prosocial Motivation (Time Point 1, Collected from Employees)

In order to assess the degree of prosocial motivation, we utilized five items from Grant and Sumanth [49]. The sample items were, “I get energized by working on tasks that have the potential to benefit others”, “It is important to me to have the opportunity to use my abilities to benefit others”, and “I prefer to work on tasks that allow me to have a positive impact on others”. The Cronbach’s alpha value is 0.85.

#### 3.2.3. Meaningfulness of Work (Time Point 2, Collected from Employees)

In order to evaluate the degree of employee meaningfulness of work, we utilized five items of the meaningfulness of work scale, which was used in previous studies [19,50]. The sample items were (a) “The work that I do is meaningful”; (b) “The work that I do makes the world a better place”; and (c) “My work is one of the most important things in my life”. The Cronbach’s alpha value is 0.86.

#### 3.2.4. Depression (Time Point 3, Collected from Employees)

The degree of depression was measured by using the 10 items of the Center for Epidemiologic Studies Depression Scale (CES-D)-10 [51]. This scale consists of major components of depression, including hopefulness, fear, loneliness, unhappiness, attention deficit, and sleep disorder. The sample items were “I felt hopeful about the future”, “I felt lonely”, “I could not “get going”, and “I felt depressed”. The Cronbach’s alpha value is 0.94.

#### 3.2.5. Control Variables

By relying on extant studies [15,16,17], the dependent variable of this research—depression—was controlled by several variables including employees’ tenure, gender, position, and education. These were gathered at the first time point.

### 3.3. Analytical Approach

Initially, a frequency analysis was undertaken to scrutinize the demographic characteristics of the respondents. Subsequently, a Pearson correlation analysis was conducted utilizing SPSS 26 (IBM Corp., Armonk, NY, USA) software to ascertain the relationships among the variables under study. Consistent with Anderson and Gerbing’s [52] guidelines, a bifurcated process incorporating the measurement and the structural model was engaged. Confirmatory factor analysis (CFA) was employed to validate the measurement model. Following this, the structural model was assessed by deploying a moderated mediation model analysis with the maximum likelihood (ML) estimator facilitated through the AMOS 26.0 software.

In this study, a spectrum of goodness-of-fit indices, including the comparative fit index (CFI), the Tucker–Lewis index (TLI), and the root mean square error of approximation (RMSEA), were harnessed to assess the suitability of diverse model fit indices. Prevailing research posits that CFI and TLI values greater than 0.90 and an RMSEA value under 0.06 are considered acceptable [53]. Subsequently, a bootstrapping analysis was carried out to determine the statistical significance of the indirect effect [54]. Ultimately, we carried out a bootstrapping analysis with a 95% bias-corrected confidence interval (CI) to validate our mediation hypothesis. This analysis proves critical in assessing the significance of the indirect mediation effect. A CI that does not encompass zero (0) denotes that the indirect effect holds statistical significance at a 0.05 level [54].

## 4. Results

### 4.1. Descriptive Statistics

The main factors of this paper (CSR, prosocial motivation, meaningfulness of work, and depression) are significantly related. Table 2 shows the result of the correlation analysis.

### 4.2. Measurement Model

In order to ascertain the discriminant validity of the primary research variables (CSR, prosocial motivation, meaningful work, and depression), we executed a confirmatory factor analysis (CFA) for all items by evaluating the goodness of fit of the measurement model. Particularly, we juxtaposed our postulated model, a four-factor model (CSR, prosocial motivation, meaningful work, and depression), with various alternate models, such as the three-, two-, and one-factor models, by undertaking a sequence of chi-square difference tests.

Primarily, the postulated four-factor model exhibited a robust and acceptable fit (χ2 (df = 125) = 169.201; CFI = 0.979; TLI = 0.974; RMSEA = 0.041). Subsequently, we conducted a series of chi-square difference tests by comparing the four-factor model with a three-factor model (χ2 (df = 128) = 408.237; CFI = 0.865; TLI = 0.838; RMSEA = 0.101), a two-factor model (χ2 (df = 130) = 570.883; CFI = 0.787; TLI = 0.750; RMSEA = 0.126), and a one-factor model (χ2 (df = 131) = 1236.399; CFI = 0.467; TLI = 0.378; RMSEA = 0.199). The chi-square difference tests showed that the four-factor model was better than others. Thus, this finding indicates that our four research variables have an appropriate degree of discriminant validity.

### 4.3. Structural Model

In the present investigation, we crafted a moderated mediation model encapsulating both mediation and moderation architectures within the correlation between CSR and depression. Within the mediation framework, the connection between CSR and depression is mediated by the magnitude of an employee’s perceived meaningfulness of work. Conversely, within the moderation framework, prosocial motivation is depicted as a buffering entity positively moderating the enhancing influence of CSR on the sense of meaningfulness derived from work.

Subsequently, an interaction term was generated through the multiplication of two central components, specifically CSR and prosocial motivation, inherent in the moderation architecture. This multiplication was preceded by the centering of each variable around their respective means in an attempt to mollify the adverse influence of multi-collinearity. This centering process bolsters the validity of the moderation analysis by attenuating the extent of multi-collinearity between the variables, thus reducing the potential degradation of the correlations [55].

In order to examine the potential bias engendered by multi-collinearity, we calculated the variance inflation factors (VIFs) and tolerances [55]. The resulting VIF values for CSR and prosocial motivation were 1.090 and 1.090, respectively. Additionally, the computed tolerance values were 0.918 and 0.918, correspondingly. These results, characterized by VIF values below 10 and tolerance values exceeding 0.2, propose that the variables CSR and prosocial motivation are largely unhampered by the issue of multi-collinearity.

#### 4.3.1. The Results of the Mediation Analysis

In order to identify the optimal mediation model, a comparison was made between the full mediation model and the partial mediation model, facilitated through a chi-square difference test. The full mediation model mirrors the partial mediation model except for the direct trajectory from CSR to depression. Both the full mediation model (χ2 = 223.035 (df = 130), CFI = 0.950, TLI = 0.934, and RMSEA = 0.058) and the partial mediation model (χ2 = 223.028 (df = 129), CFI = 0.949, TLI = 0.933, and RMSEA = 0.058) demonstrated acceptable fit indices. However, the chi-square difference test between the models (Δχ2 [1] = 0.007, nonsignificant) indicated that the full mediation model was superior. This finding suggests that CSR’s influence on depression is likely to be indirect, possibly through the mediating influence of the meaningfulness of work.

Control variables (tenure, gender, education, and position) were incorporated into the research model to adjust for the dependent variable, depression. The results revealed that all control variables were statistically insignificant.

The research model, inclusive of control variables, demonstrated a nonsignificant correlation between CSR and employee depression (β = −0.008, *p* > 0.05), thereby refuting Hypothesis 1. For Hypothesis 1, the path coefficient from CSR to depression was within the “partial” mediation model, which was considered inferior to the full mediation model and was subsequently rejected. This result aligns with the notion that the fit indices of full mediation surpass those of partial mediation. Given the results of the chi-square difference test between the full and partial mediation models, coupled with the nonsignificant path coefficient value, we surmise that Hypothesis 1 is not supported. In essence, CSR is likely to wield an “indirect” influence on depression through the mediating effect of various mediators (such as the meaningfulness of work) rather than a direct one.

**Figure 2:**
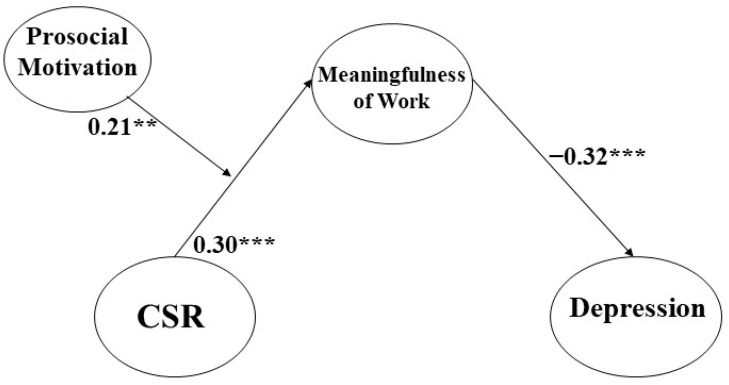
The final result of our research model with standardized values; ** *p* < 0.01, *** *p* < 0.001.

CSR was found to have a significant and positive association with employees’ sense of meaningful work (β = 0.30, *p* < 0.001), thereby supporting Hypothesis 2. Additionally, the sense of meaningfulness of work demonstrated a significant and negative correlation with depression (β = −0.32, *p* < 0.001), lending support to Hypothesis 3 (please refer to Table 3 and Figure 2).

#### 4.3.2. Bootstrapping

In order to scrutinize the mediating function of work’s meaningfulness within the connection between CSR and depression (Hypothesis 4), we utilized a bootstrapping procedure with a representative sample of 10,000 [54]. An indirect mediation effect will be deemed significant at the 5% level if the 95% bias-corrected confidence interval (CI) for this particular effect does not include 0 [54].

The results indicated that the bias-corrected CI for the average indirect effect excluded 0 (95% CI = [−0.150, −0.018]). This infers that the indirect mediation effect of work’s meaningfulness was statistically meaningful, hence validating Hypothesis 4. A detailed depiction of the direct, indirect, and total effects of the trajectories from CSR to depression is demonstrated in Table 4.

#### 4.3.3. The Result of the Moderation Analysis

**Figure 3:**
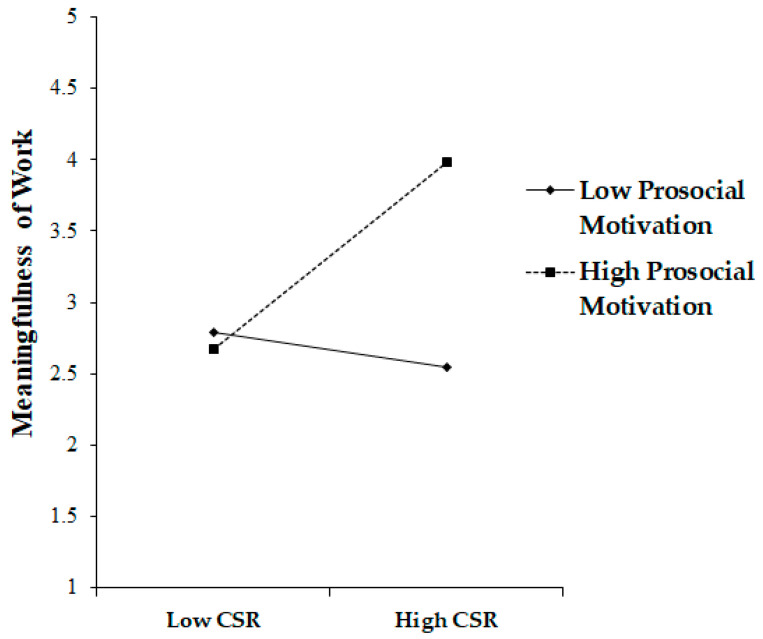
Moderating effect of prosocial motivation in the CSR–Meaningfulness of Work link.

We scrutinized the moderation influence of prosocial motivation on the interconnection between CSR and the meaningfulness of work. For this purpose, we instigated a mean-centering operation to construct an interaction term. The coefficient figure of the interaction term (β = 0.21, *p* < 0.001) was statistically consequential. This outcome suggests that prosocial motivation optimistically moderates the link between CSR and the meaningfulness of work by functioning as an enhancer. Furthermore, it indicates that when the degree of prosocial motivation is elevated, the augmented impact of CSR on the meaningfulness of work is amplified, corroborating Hypothesis 5 3 (please refer to Figure 3).

## 5. Discussion

Using a three-stage, time-separated dataset, we discovered that the significance employees attribute to their work functions as a mediator in the relationship between CSR and employees’ depression. Additionally, we uncovered that employees’ prosocial motivation positively influences the CSR-meaningfulness of work link. The ensuing section articulates the theoretical and practical consequences of this study, acknowledges the limitations, and proposes directions for future research. 

### 5.1. Theoretical Implications

We believe that the theoretical implications outlined in this study will contribute positively to the literature on CSR. First, we investigated the influence of CSR on employees’ mental health (i.e., depression), which has been relatively understudied by CSR scholars despite its importance to an organization. The literature survey reveals that few works have delved into the association between CSR and employees’ mental health, including depression, anxiety, and burnout at work [2,3,8,11]. Considering that employees’ mental health significantly influences their perceptions, attitudes, behaviors, and even job performance, either directly or indirectly [2,3,8,11,12,13,14,15,16,17], our initiative to explore this relationship and demonstrate the effect of CSR on their depression is an essential endeavor.

Second, the research aimed to explore the intermediating process (mediator) and its contingent factor (moderators) of the connection between CSR and depression, looking at this from the perspective of the employees’ ‘sense-making’ process. Given that the processes of interpreting and ‘making sense’ typically shape employees’ perceptions and attitudes towards CSR initiatives, our discoveries about the significance of work and prosocial motivation—considered to be key variables related to sense-making (serving as the mediator and moderator of the relationship)—may aid in broadening the CSR literature [2,3,8,11].

Specifically, we discovered that the motivational characteristics of employees, such as prosocial motivation, profoundly influence how CSR initiatives affect the sense of meaningful work. The outcomes of our research revealed that the positive influence of CSR on the perceived significance of work is intensified by a high level of prosocial motivation, indicating that the degree of prosocial motivation is a pivotal contingent factor in translating ethical efforts (i.e., CSR) into augmented employees’ positive perceptions (i.e., the significance of work).

Third, we scrutinized the intermediating and contingent variables of the CSR-depression correlation by utilizing factors associated with psychology, such as the perceived significance of work and prosocial motivation [2,8,21]. Positive psychology tends to interpret various organizational phenomena from the angle of positive procedures, characteristics, and outcomes instead of negative ones [21]. Given that a ‘real’ organization encompasses both positive and negative factors, it could be insightful to explore the intermediating processes and contextual variables from the perspective of positive psychology.

Fourth, South Korea’s economic transformation in the second half of the 20th century, often referred to as the “Miracle on the Han River,” has deeply influenced its work culture. Rapid industrialization and economic growth have fostered a work environment characterized by hard work, diligence, and commitment. South Koreans typically prioritize group cohesion and harmony, often valuing collective interests over individual ones. This communal emphasis is rooted in Confucian ideals that emphasize respect for authority, loyalty to the company, and maintaining harmony within the group [27,56]. With globalization and increased international trade, South Korean corporations have become more aware of the significance of CSR for both ethical and strategic reasons. Initially, CSR in South Korea was largely philanthropic, with corporations making charitable donations or funding community projects. In recent years, however, there has been a shift towards a more comprehensive approach to CSR, encompassing environmental, social, and governance factors. However, the understanding and implementation of CSR can sometimes be superficial, driven more by image-building and public relations than by deep-seated ethical considerations [25,27]. South Korea also faces significant challenges related to mental health, with depression being a major concern [15,29]. Despite having a robust healthcare system, mental health has historically been overshadowed by social stigma and lack of awareness. Depression rates have been relatively high, and the country has struggled with high suicide rates compared to other OECD countries [29]. The rigorous work culture, societal pressures, and, often, the lack of a work-life balance have contributed to stress and mental health problems among the working population [15,24]. By understanding these cultural nuances, readers can better appreciate the significance of research on the relationship between CSR and depression in the South Korean context. This background will provide a richer context for findings and insights.

Finally, establishing a clear link between depression and the work environment is pivotal to grounding research in the ‘CSR-depression link’. We, therefore, suggest a deeper reflection on how depression is related to work and its environment. Depression, a prevalent mental disorder characterized by persistent feelings of sadness, hopelessness, and a lack of interest or pleasure in activities, has many causes, ranging from biological factors to environmental influences. Among these, the work environment is a major contributor to the onset and exacerbation of depressive symptoms.

Prolonged exposure to work-related stressors, such as an excessive workload, role ambiguity, conflicting job demands, and lack of control over tasks, can trigger or exacerbate depressive symptoms. Chronic stress has physiological repercussions that can disrupt the balance of neurotransmitters in the brain, which are vital for mood regulation. In addition, the inability to balance professional and personal life can lead to emotional exhaustion, which, over time, can lead to feelings of detachment, hopelessness, and other depressive symptoms. A workplace that lacks collegiality, appreciation, or supportive supervision can create an environment of isolation. Feeling undervalued or unrecognized can gradually erode an individual’s sense of self-worth, contributing to depression. In addition, in an era of rapid economic change, the looming threat of job loss or sudden organizational change can be a substantial psychological burden, contributing to anxiety and depressive feelings.

Moreover, the ‘CSR-depression link’ posits a relationship between a company’s commitment to ethical, social, and environmental responsibility (CSR) and the mental well-being (specifically, depression levels) of its employees. Several factors may influence this link. First, employees often view CSR initiatives as an indication that the company cares not just about profits but also about the well-being of society and its employees. This can increase perceived organizational support, which, in turn, has been linked to reduced depressive symptoms. CSR initiatives can make employees feel that their work contributes to a greater social good, thereby increasing the intrinsic meaningfulness of their role. A sense of purpose and meaning in work can act as a buffer against depressive feelings. Second, employees’ pride in and attachment to an organization can be fortified by effective CSR initiatives, creating a sense of belonging. This sense of identity and belongingness can reduce feelings of isolation, a major contributor to depression. Third, CSR often involves teamwork and cross-departmental collaboration on community or environmental projects. This can foster camaraderie among colleagues and reduce feelings of isolation at work, a major trigger for depression.

In essence, the ‘CSR-depression link’ underscores the potential of CSR initiatives to not only enhance a company’s external image but also to have a tangible impact on the mental well-being of its internal stakeholders—the employees. A comprehensive exploration of this link necessitates an understanding of how the various facets of CSR resonate with the psychological needs of employees and how they shape perceptions, attitudes, and overall mental health in the context of their work environment. This detailed reflection should provide clarity on the complex relationship between depression, the work environment, and CSR and offer a solid foundation for your exploration of the ‘CSR-depression link’.

### 5.2. Practical Implications

The present research may have practical implications for a firm’s senior leadership teams. First, based on the evidence that CSR reduces the extent of employee depression, we suggest viewing CSR as an effective and efficient “investment” rather than just a ‘moral duty and expense’. Given that employees’ levels of depression or depressive symptoms significantly influence their mental/physical health and job performance [15,16,17], lowering the severity of depression through CSR initiatives might be a plausible strategy for organizations. Therefore, senior leadership teams should endeavor to communicate with employees about CSR and provide incentives that bolster their involvement. This is one potential way through which the upper management can cultivate a CSR-centric culture in an organization.

Second, for CSR initiatives to yield positive impacts within an organization, it is necessary for senior management teams to appreciate the importance of employees’ sense-making processes. It is improbable that employees will accept organizational policies, systems, and activities passively. They actively interpret and strive to manipulate their environmental factors to their advantage. Additionally, employees’ perceptions and attitudes towards CSR activities are likely to be significantly shaped by their sense-making processes or interpretations [18,20]. Therefore, it is incumbent upon the top management team to monitor and comprehend how employees make sense of and interpret CSR practices. 

Particularly, for the successful execution of CSR, upper management must acknowledge the pivotal role that the motivational traits of employees (i.e., prosocial motivation) assume. The beneficial impact of CSR on the meaningfulness of employees’ work may diminish if their level of prosocial motivation is low. This suggests that the extent of prosocial motivation might serve as a dependable indicator of whether CSR positively influences the meaningfulness of work.

Third, by discerning the mediating mechanism that underpins the association between CSR and depression, the senior management teams are empowered to forecast the efficacy of the organization’s CSR initiatives. This research has illuminated the fact that the extent of meaningfulness that employees attribute to their work mediates the relationship between CSR and depression. It advocates for the senior management teams to scrupulously evaluate and observe alterations in the level of meaningfulness that employees attribute to their work when they aspire to alleviate the degree of depression through CSR initiatives. For instance, if there is no increase in the meaningfulness of work for employees even after the implementation of CSR, such ethical endeavors might prove ineffective. By observing these changes, the senior management teams would gather indispensable data pertaining to the efficacy of CSR.

### 5.3. Limitations and Suggestions for Future Studies

The following section elucidates some limitations of the present paper. First, this research did not sufficiently encapsulate the cultural discrepancies between Eastern and Western societies. These variances might significantly influence the way employees comprehend and interpret CSR initiatives. Western societies typically tend to underscore the significance of social responsibilities, such as CSR or business ethics. Consequently, Western employees may exhibit greater sensitivity to CSR activities in comparison to their Eastern counterparts [27,56]. This research solicited data solely from employees working in South Korean firms; hence, the interpretations and applications of its findings should be approached cautiously in organizations with diverse cultural contexts [56,57]. Despite recognizing the potential universality of CSR principles [56,58], we cannot dismiss the impact of cultural disparities. Consequently, we advocate that future inquiries address this concern.

More specifically, the intersection of culture and CSR remains a fertile ground for scholarly exploration. While substantial research has focused on the mechanisms and outcomes of CSR, a nuanced understanding of how cultural contexts influence employees’ perceptions and interpretations of CSR initiatives remains underdeveloped. Future research in this area should prioritize the exploration of cultural differences between Eastern and Western societies to comprehend the intricate tapestry of beliefs, values, and practices that shape organizational behavior. In order to be specific, a starting point would be to develop comparative studies that juxtapose how Eastern and Western societies perceive and interpret CSR initiatives. Such studies should aim to (1) document the historical, philosophical, and socio-economic foundations of CSR in different cultural contexts, offering insights into how different societies have traditionally approached concepts related to corporate responsibility and ethics. (2) Explore how core cultural values (e.g., individualism vs. collectivism and high vs. low power distance) influence employees’ expectations of their companies in terms of social and environmental responsibility. (3) Understand how cultural orientations shape the definitions of what constitutes ‘responsible’ corporate behavior, the expected boundaries of corporate intervention in societal issues, and the weight given to different CSR pillars.

Future research should also explore the role of cultural mediators—elements that may influence the transmission and interpretation of cultural values related to CSR. For instance, (1) how do national education systems, known to be both transmitters and shapers of societal values, address corporate ethics and responsibility in their curricula? (2) How do media representations of CSR, which vary across countries, affect societal and employee perceptions?

Given the increasing globalization of business, how can companies utilize cross-cultural training to harmonize CSR perceptions among their geographically diverse workforces? Research could explore (1) the design and effectiveness of training programs aimed at bridging cultural gaps in CSR understanding and (2) strategies for multinational corporations to balance global CSR standards with local cultural nuances.

The evolving landscape of CSR necessitates a thorough cultural lens to comprehend the myriad of ways in which societal values and norms shape CSR interpretations. By addressing the aforementioned research avenues, scholars can unravel the complexities of the CSR-culture nexus, enabling companies to more effectively navigate their CSR journeys in culturally diverse environments.

Second, this study could not implement an objective measure to assess the extent of CSR activities. The pre-existing studies on CSR suggest that the subjective indicators of CSR, such as employee perceptions of the degree of CSR activities, aptly mirror the actual phenomena of CSR [59]. Nonetheless, we propose that future investigations should employ both subjective and objective CSR measures and examine their distinct influences.

More specifically, as the importance of CSR has grown, so has the urgency to measure and assess its activities. In pursuit of an accurate and holistic assessment of CSR activities, it is pivotal that future studies adopt a more nuanced approach, incorporating both subjective and objective metrics. Subjective measures, typically rooted in employee perceptions, opinions, or attitudes, offer invaluable insights into the internal resonance of CSR initiatives. Employee perceptions reflect the alignment (or lack thereof) between a company’s communicated CSR goals and its perceived commitment and delivery. 

Objective measures, on the other hand, bring quantitative rigor to CSR assessment. They offer tangible metrics, such as the amount of waste reduced, the percentage of energy generated from renewable sources, or the funds allocated to community development projects. The objective measures can also provide a standardized assessment that can be compared over time with competitors or with industry benchmarks. The measures would also counterbalance the potential biases or inaccuracies inherent in subjective perceptions, ensuring that evaluations are grounded in empirical reality.

A discrepancy between objective CSR performance and subjective employee perceptions can indicate communication gaps and highlight areas where internal CSR communication strategies need to be strengthened. While employees are crucial internal stakeholders, objective measures address a wider range of external stakeholders, including investors, regulators, and NGOs. A balanced approach to measurement addresses the information needs of different stakeholder groups.

Thus, in the dynamic landscape of CSR, where corporate actions are under constant scrutiny and where authenticity and impact are paramount, a comprehensive assessment approach is non-negotiable. By harmonizing subjective perceptions with objective realities, future studies can pave the way for a more transparent, accountable, and effective implementation of CSR initiatives.

Third, despite utilizing three-wave time-lagged data, this study is not exempt from the problem of common method bias (CMB). In order to counter this, we also conducted Harman’s single-factor test, a popular technique employed to estimate the extent of CMB [60]. The result suggested that the CMB issue was minimal, as only 26.99% of the covariance could be attributed to a single factor. However, we recommend that future research should corroborate these findings using multiple data sources.

## 6. Conclusions

This study explored the effect of CSR on employee depression. The findings elucidated that CSR attenuates the severity of employee depression via the mediating influence of meaningful work. Additionally, prosocial motivation functions as a beneficial moderator in the relationship between CSR and the significance attributed to work. The outcome suggests that the meaningfulness of work is the foundational process through which CSR activities translate into mitigating employee depression. Furthermore, prosocial motivation operates as an augmenting variable, enhancing the positive impact of CSR. Despite the constraints present in the current research, we are confident that our findings can make a substantial contribution to the literature surrounding CSR and depression [61,62].

## Figures and Tables

**Table 1:** Descriptive characteristics of the sample.

Characteristic	Percent
Gender	
Male	51.9%
Female	48.1%
Age (years)	
20–29	12.6%
30–39	35.5%
40–49	33.2%
50–59	18.7%
Education	
Below high school	8.4%
Community college	16.8%
Bachelor’s degree	62.1%
Master’s degree or higher	12.6%
Occupation	
Office worker	74.9%
Profession (Practitioner)	7.7%
Production worker	4.8%
Administrative positions	4.8%
Public official	3.9%
Sales and Service	2.9%
Education	0.5%
Freelance	0.5%
Position	
Staff	22.0%
Assistant Manager	18.7%
Manager or deputy general manager	35.5%
Department/general manager or director and above	23.8%
Tenure (years)	
Below 5	45.8%
5–10	27.6%
11–15	14.9%
16–20	5.2%
21–25	2.8%
Above 26	3.7%
Industry Type	
Manufacturing	24.6%
Construction	14.0%
Wholesale/Retail business	12.1%
Health and welfare	10.1%
Information service and telecommunications	9.7%
Services	10.1%
Education	6.7%
Financial/insurance	3.4%
Consulting and advertising Others	0.5%
Others	8.7%

**Table 2:** Correlation between variables.

	Mean	S.D.	1	2	3	4	5	6	7
1. Gender_T2	1.47	0.50	-						
2. Education_T2	2.73	0.79	−0.20 **	-					
3. Tenure_T2	7.91	7.57	−0.31 **	−0.01	-				
4. Position_T2	3.04	1.62	−0.47 **	0.19 **	0.31 **	-			
5. CSR_T1	3.20	0.62	−0.16 *	0.001	0.19 **	0.10	-		
6. PM_T1	3.22	0.59	−0.11	0.06	0.12	0.17 *	0.29 **	-	
7. MoW_T2	3.24	0.59	−0.20 **	0.11	0.09	0.32 **	0.43 **	0.35 **	-
8. Dep_T3	3.71	0.56	0.05	−0.07	−0.13	−0.07	−0.17 *	−0.05	−0.30 **

Notes: * *p* < 0.05. ** *p* < 0.01. S.D. means standard deviation, CSR means corporate social responsibility, PM means prosocial motivation, MoW means meaningfulness of work, and Dep indicates depression or depressive symptoms. As for gender, males are coded 1 and females 2. As for positions, a general manager or higher is coded as 5, deputy general manager and department manager 4, assistant manager 3, clerk 2, and others below clerk as 1. As for education, the “below high school diploma” level is coded 1, the “community college” level is coded 2, the “bachelor’s” level is coded 3, and the “master’s degree or more” level is coded 5.

**Table 3:** Results of the structural model.

Hypothesis	Path (Relationship)	Unstandardized Estimate	SE	Standardized Estimate	Supported
1	CSR -> Depression	−0.005	0.059	−0.008	No
2	CSR -> Meaningfulness of Work	0.267	0.072	0.300 ***	Yes
3	Meaningfulness of Work -> Depression	−0.249	0.064	−0.320 ***	Yes
5	CSR × Prosocial Motivation	0.389	0.123	0.212 **	Yes

Note: ** *p* < 0.01. *** *p* < 0.001. Estimate indicates standardized coefficients. S.E. means standard error.

**Table 4:** Direct, indirect, and total effects of the final research model.

Model (Hypothesis 4)	Direct Effect	Indirect Effect	Total Effect
CSR → POS → Organizational Commitment → Knowledge-sharing Behavior	0.229	0.047	0.277

All values are standardized. CSR: corporate social responsibility; POS: perceived organizational support.

## Data Availability

New data were created and analyzed in this study. Data sharing is not applicable to this article.
